# Subjective Symptoms and Disease Activity Related to Serum Zinc Concentration in Primary Sjögren’s Syndrome

**DOI:** 10.3390/jcm13164672

**Published:** 2024-08-09

**Authors:** Kumiko Akiya, Masahiro Nishihara, Yosuke Nagasawa, Noboru Kitamura, Hisataka Kitano, Jun Shoji, Yu Iwabuchi, Hiroyuki Hao, Hideki Nakamura

**Affiliations:** 1 Division of Hematology and Rheumatology, Department of Medicine, Nihon University School of Medicine, Tokyo 173-8610, Japan; akiya.kumiko@nihon-u.ac.jp (K.A.); nishihara.masahiro@nihon-u.ac.jp (M.N.); nagasawa.yosuke@nihon-u.ac.jp (Y.N.); kitamura.noboru@nihon-u.ac.jp (N.K.); 2 Division of Oral and Maxillofacial Surgery, Department of Medicine, Nihon University School of Medicine, Tokyo 173-8610, Japan; kitano.hisataka@nihon-u.ac.jp; 3 Division of Ophthalmology, Department of Visual Sciences, Nihon University School of Medicine, Tokyo 173-8610, Japan; shoji.jun@nihon-u.ac.jp; 4 Department of Radiology, Keio University School of Medicine, Tokyo 160-8582, Japan; iwabuchi@rad.med.keio.ac.jp; 5 Division of Human Pathology, Department of Pathology and Microbiology, Nihon University School of Medicine, Tokyo 173-8610, Japan; hao.hiroyuki@nihon-u.ac.jp

**Keywords:** zinc deficiency, Sjögren’s syndrome, ESSDAI, disease activity

## Abstract

**Background/Objectives**: We examined the frequency of zinc deficiency in patients with Sjögren’s syndrome (SS) and the relationship between zinc deficiency and each of the subjective symptoms and disease activity. **Methods**: We enrolled 164 patients aged ≥ 20 years with primary SS (pSS) based on the revised diagnostic criteria of the Ministry of Health, Labor and Welfare (1999) and 144 patients with RA diagnosed according to the ACR/EULAR classification criteria for RA (2010) as a comparison group. Subjective symptoms were confirmed using an original questionnaire, and disease activity was determined using the European League Against Rheumatism Sjögren’s Syndrome Disease Activity Index (ESSDAI). The serum zinc concentrations were measured in both SS and RA patients. **Results**: The rate of zinc deficiency in the SS group was 26.1%, significantly higher than that in the RA group (7.6%). The rate of zinc deficiency was significantly higher in the pSS group compared with Japanese health checkup recipients reported in the literature. The mean serum zinc concentration in primary SS was 60.6 ± 7.3 µmol/L in the high disease activity group with an ESSDAI of ≥5 points, which was significantly lower than the concentration of 69.7 ± 10.2 µmol/L in patients with an ESSDAI of ≤4 points. **Conclusions**: The frequency of zinc deficiency was higher in patients with pSS than in patients with RA. Disease activity was also higher in patients with zinc deficiency, suggesting an association between zinc concentration and organ involvement in pSS.

## 1. Introduction

Sjögren’s syndrome (SS) is a representative autoimmune disease characterized by exocrine dysfunction and the appearance of anti-Ro/SS-A and La/SS-B antibodies, which are produced by T cell activation and subsequent B cell proliferation [1,2,3].

Zinc deficiency generally reduces protein synthesis in the body, since zinc is required for the production of more than 300 enzymes, including deoxyribonucleic acid (DNA) polymerase and alkaline phosphatase [4,5,6]. Zinc deficiency has been associated with symptoms such as dysgeusia, decreased appetite, susceptibility to infection, and delayed wound-healing [7,8]. Zinc deficiency has also been related to life expectancy and has therefore attracted increasing attention in the context of aging societies [9,10]. Zinc is known to have important effects on immune function, oxidative stress, and apoptosis. It has been reported that zinc deficiency may affect immune abnormalities in many chronic diseases, such as malignant tumors, neurological diseases, and autoimmune diseases, leading to the production of inflammatory cytokines and increasing disease activity [11,12].

Liver cirrhosis, diabetes, and chronic kidney disease are all known to cause zinc deficiency [13,14,15]. Zinc status also plays a role in autoimmune disease, as detailed in a 2018 review of the interaction between the two conditions [16]. In that systematic review, 17 of 18 meta-analyses on rheumatoid arthritis (RA) showed significantly decreased serum or plasma levels of zinc in RA patients. In regard to SS, there has been only one report in which the mean serum Zn concentration in 31 cases of SS was 70.6 ± 13.8 µg/dL, which was significantly lower than 86.6 ± 15.9 µg/dL in 15 cases in the non-SS subjects complaining of dry eyes and dry mouth [17]. In addition, the serum zinc concentrations increased in 64 of 81 RA patients who had baseline serum zinc concentrations less than 70 µg/dL before receiving polaprezinc, a drug containing zinc for the treatment of gastric ulcers, for 6 months or longer; moreover, the C-reactive protein (CRP) levels and disease activity scores in 28 joints (DAS28) in these patients significantly improved, suggesting an impact on disease activity in RA [18].

In this study, we investigated the extent to which zinc deficiency and subclinical zinc deficiency exist in SS patients based on Japan’s Practical Guideline for Zinc Deficiency issued in 2018 by the Japanese Society of Clinical Nutrition [19], and we compare the frequencies of zinc deficiency between patients with SS and patients with RA, a condition in which zinc deficiency has frequently been reported [20,21]. We also investigate the relationship between subjective symptoms and disease activity in SS patients.

## 2. Materials and Methods

### 2.1. Patients

We enrolled patients aged 20 or older who visited the Department of Hematology and Rheumatology at Nihon University Itabashi Hospital or the Department of Rheumatology at the National Hospital Organization Tokyo Medical Center between 1 April 2018 and 30 April 2022. A total of 164 patients with primary SS diagnosed according to the revised diagnostic criteria for Sjögren’s syndrome (1999) [22] were enrolled. After excluding 8 patients who did not fulfill the criteria of the American College of Rheumatology/EULAR classification (2016) for SS, the remaining 156 patients were included in the analysis [23].

The characteristics of the enrolled primary SS patients are shown in Table 1. Then, 144 patients with RA diagnosed according to the ACR/EULAR Rheumatoid Arthritis Classification Criteria (2010) [24] and for whom measurements of serum zinc concentrations were available were also included as a control group. Patients who had both SS and RA were excluded.

We compared our data with those reported regarding zinc deficiency/marginal deficiency among Japanese subjects undergoing health checkups in Japan [25].

The main characteristics of the subjects are shown in Table 2. The serum zinc concentrations were measured using the colorimetric method at our institution (normal range: 80–130 µg/dL).

### 2.2. Clinical Assessment

We performed the following tests: a questionnaire about symptoms of dry mouth and eyes, a Saxon test, a gum test, ^99m^Tc–pertechnetate salivary gland scintigraphy, Schirmer’s test, Fluorescein staining, white blood cell count (WBC), white blood cell differential (Neutrophil, Lymphocyte), hemoglobin (Hb), platelets count (Plt), anti-Ro/SS-A antibody, and anti-La/SS-B antibody, minor salivary gland biopsy. Subjective symptoms were investigated using an original questionnaire (Supplementary Table S1), and responses were obtained on an 11-point scale, with 0 indicating no symptoms and 10 indicating the strongest symptoms. Disease activity was assessed using the European League Against Rheumatism Sjögren’s Syndrome Disease Activity Index (ESSDAI) [26].

### 2.3. Statistical Analysis

Statistical analysis: Two-sample *t*-tests. A comparison of the quantitative parameters between the two groups was performed using the non-parametric Mann–Whitney *U* test.

Discrete variables were analyzed using the Chi^2^ test. A correlation analysis was performed using the nonparametric Spearman coefficient. SPSS Statics 21 (IBM, Chicago, IL, USA) was used as statistical analysis software, and values of *p* < 0.05 were considered statistically significant.

## 3. Results

### 3.1. Comparison between Patient Groups

We compared our data with those reported regarding zinc deficiency/marginal deficiency among Japanese subjects undergoing health checkups in Japan [25]. It has been reported that no difference in mean zinc concentrations between age groups was observed in women who underwent health checkups. Therefore, we compared zinc deficiency/marginal deficiency/normal concentrations in patients with pSS to the RA and health examination groups in women only. In the pSS group, the proportion of people with normal zinc levels was significantly lower than those in the health checkup group, and the rate of zinc deficiency/marginal deficiency was significantly higher (*p* < 0.001) (Table 3).

The frequency of zinc deficiency was 26.1% (41/157 patients) in the SS and 7.6% (9/119 patients) in the RA group, with the former being significantly higher (*p* < 0.001) (Table 3). The frequency of normal zinc concentrations was 10.8% (17/157 patients) in the SS and 22.7% (27/119 patients) in the RA group, with the frequency in SS patients being significantly lower (*p* < 0.05) (Table 3).

Six of the seven men with pSS were aged 70 years or older, and the mean zinc concentration was 66.0 µg/dL. According to a graph provided in the literature, the mean zinc concentrations in men aged 70 years or older who underwent health checkups was close to 80 µg/dL, but the exact values were not given, so analysis was not possible.

### 3.2. Association with Subjective Symptoms

**Figure 1:**
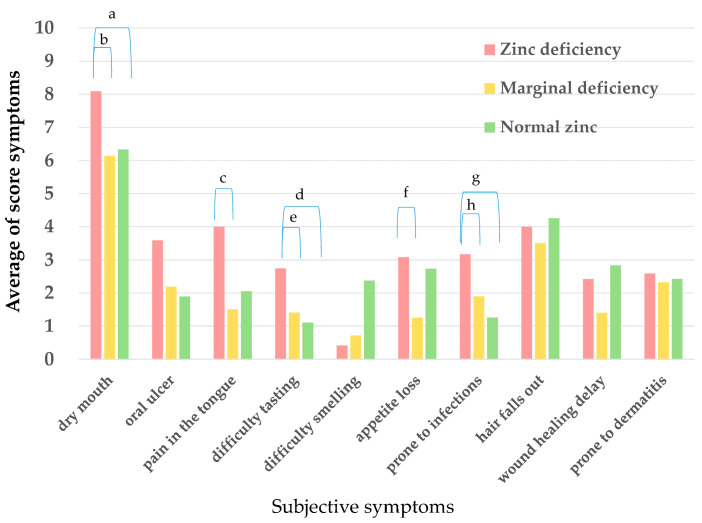
Comparison of subjective symptoms due to zinc deficiency in primary SS patients. Primary SS patients were divided into three groups: patients with zinc deficiency (Zn < 60 µg/dL), latent zinc deficiency (60 ≤ Zn < 80 µg/dL), or normal zinc levels (Z ≥ 80 µg/dL). Their subjective symptom scores (0–10) were then determined using a questionnaire. Significant at *p* < 0.05 according to Mann–Whitney *U*-test. a: *p* = 0.041, b: *p* = 0.007, c: *p* = 0.005, d: *p* = 0.030, e: *p* = 0.040, f: *p* = 0.007, g: *p* = 0.028, h: *p* = 0.039.

In relation to subjective symptoms, symptoms such as dry mouth, tongue pain, difficulty in tasting, and propensity for infections tended to be more severe in SS patients with zinc deficiency than in those with normal zinc levels (*p* < 0.05) (Figure 1).

### 3.3. Association with Disease Activity

When comparing the mean zinc concentrations between patients with low disease activity (ESSDAI < 5) and those with moderate to high disease activity (ESSDAI ≥ 5), the mean zinc values were significantly lower in the latter group (69.7 ± 102 vs. 60.6 ± 7.3 µg/dL; *p* < 0.05) (Table 4). A weak negative correlation with a correlation coefficient of −0.3598 was observed between the serum zinc concentration and the ESSDAI score in primary SS (*p* < 0.001).

Among the laboratory items included in the ESSDAI, weak negative correlations were observed between serum zinc concentrations and platelet and Hb concentrations, but no associations were found for neutrophil or lymphocyte counts, or levels of IgG or C3. No significant correlation was found between serum zinc and serum albumin concentrations in patients with primary SS.

### 3.4. Association with Extraglandular Manifestations

The mean serum zinc concentrations for each extra-glandular manifestation in the pSS group were as follows: malignant lymphoma: 56.7 ± 10.5 µmol/L; peripheral neuropathy: 59.0 ± 5.0 µmol/L; interstitial pneumonia: 60.4 ± 6.0 µmol/L; interstitial nephritis: 62.3 ± 10.5 µmol/L; articular manifestations: 64.0 ± 7.1 µmol/L; and skin manifestations: 67.2 ± 7.1 µmol/L, with a significant difference only being observed between malignant lymphoma and skin symptoms (*p* < 0.033). The mean serum zinc concentrations for each autoantibody were as follows: anti-Ro/SS-A antibody positive group: 67.1 ± 9.4 µmol/L; negative group: 69.4 ± 14.3 µmol/L; anti-La/SS-B antibody positive group: 66.6 ± 8.6 µmol/L; negative group: 67.8 ± 10.8 µmol/L. No significant differences were observed.

## 4. Discussion

According to a previous report on zinc deficiency observed during health checkups in Japan, there was no difference in the average serum zinc concentration among women by age group. Because there were only a small number of men with SS, it was difficult to analyze, so we targeted women. We compared pSS, RA, and serum zinc concentrations among health checkup participants. The results revealed that zinc deficiency was more common in pSS patients than in RA patients, and zinc deficiency was significantly more common in pSS patients than in the health checkup participants [25]. Subjective symptoms included dry mouth, pain in the tongue, difficulty in tasting, and propensity for infections. It was suggested that these symptoms may have been at least partly due to zinc deficiency. Zinc contributes to various homeostatic functions, including growth, wound healing, immune functions, skin metabolism, taste and olfaction, saliva secretion, glucose metabolism, and lipid metabolism [27]. Consequently, a deficiency of zinc can lead to infections, delayed wound healing, anorexia, and growth disorders, and has also been associated with many diseases, especially immune disorders [4].

In relation to disease activity, a weak correlation between serum zinc concentration and ESSDAI was observed, and the serum zinc concentration was significantly lower in the patients with moderate to high disease activity (ESSDAI ≥ 5) (Table 3). Looking at each item of ESSDAI, a weak negative correlation was observed between serum zinc concentration and the platelet count and Hb value.

There was no significant correlation between serum zinc concentrations and serum albumin concentrations in SS patients, suggesting that the zinc deficiency in our patients was attributable to mechanisms other than a malnutrition-induced increase in urinary zinc excretion or Alb deficiency.

A previous study found that 64 of 81 RA patients with serum zinc levels < 70 µg/dL who took polaprezinc (a zinc preparation) for 6 months or more exhibited significant increases in serum zinc concentrations and significant improvements in CRP and DAS28 [18]. In another study in which 44 patients with rheumatoid arthritis received either zinc sulfate capsules (50 mg/day; *n* = 22 patients) or placebo and standard care (*n* = 22 patients), those taking zinc showed significant improvements in both clinical symptoms and laboratory tests after one month of treatment compared to those taking the placebo [28]. In collagen-induced arthritis and experimental autoimmune encephalomyelitis, zinc-supplemented mice exhibit less severe disease than normal water-fed mice [29]. The administration of polaprezinc (zinc preparation) to ulcerative-colitis-induced mice improves clinical symptoms [30]. Furthermore, there are reports that dietary zinc is useful for controlling IgA nephropathy [31]. Zinc concentrations are low in pemphigoid [32], but significantly higher in multiple sclerosis, suggesting different mechanisms [33].

Zinc functions as a modulator of the immune response and is regulated by several transporters and regulators. Zinc deficiency affects the cells involved in both innate and adaptive immunity. These cells include monocytes, polymorphonuclear cells, natural killer cells, T cells, and B cells. T cell function and the balance between T helper cell subsets are particularly sensitive to changes in zinc status. Chronic zinc deficiency increases the production of proinflammatory cytokines and is known to influence the outcome of many inflammatory diseases, including rheumatoid arthritis [34]. Zinc deficiency is also known to influence the balance of the helper T cell subset, while chronic zinc deficiency has been shown to cause the overproduction of proinflammatory cytokines in autoimmune diseases, including RA [34].

Normally, the signaling molecule STAT3 is activated by IL-6 stimulation. It was found that zinc has the ability to suppress the differentiation of effector Th subsets. In addition, it was observed that the number of Th17 cells in the lymph nodes decreased in mice fed with zinc water. These results suggest that the suppressive effect of zinc supplementation on autoimmune diseases may be due to the inhibition of the differentiation of Th17 cells, which produce the cytokine IL-17. As a result, zinc suppresses the development of inflammatory diseases by suppressing the IL-6/STAT3-signaling pathway [29].

In our present analysis of the relationship between serum zinc concentrations and serum albumin concentrations in patients with primary SS, only 8 of 116 cases (6.9%) with a zinc concentration of less than 80 µg/dL had low albumin.

The causes of zinc deficiency are considered to be as follows: (1) a lack of zinc intake (malnutrition, etc.); (2) the decreased absorption of zinc from the small intestine (chronic liver disease, inflammatory bowel disease, etc.); (3) decreased zinc-binding protein (of 66% Alb, 32% is known to bind to α-macroglobulin; as an example, it has been reported that zinc and Alb are correlated in chronic liver disease and Crohn’s disease); (4) increased zinc excretion (long-term use of drugs with chelating action, diabetes, renal disease, hemolytic anemia, etc.); and (5) increased demand (pregnancy, etc.) [19]. In this study, no correlation was found between serum zinc and Alb concentrations, suggesting that insufficient zinc intake due to malnutrition and decreased concentrations of zinc-binding protein are unlikely to be the cause. Other possible causes include decreased zinc absorption, increased zinc excretion, and decreased serum concentrations due to uptake into lymphocytes. Further detailed research is warranted.

There were some limitations to this study. First, the number of cases was not large; thus, in future studies, more cases will be needed. Second, since this study was retrospective, it will be necessary to conduct a prospective study to examine whether zinc administration improves disease activity. Third, we were unable to investigate whether there is a relationship between disease activity and zinc concentration in RA. However, further analysis is needed to determine whether the relationship between disease activity and zinc deficiency is similarly observed in collagen diseases other than SS. In addition, the reason why zinc deficiency is common in pSS has not been proven at the basic medical level, and the reason why zinc deficiency is more common in pSS than in RA has not been elucidated.

## 5. Conclusions

We observed a higher incidence of low serum zinc concentration in patients with primary SS compared to health checkup recipients. In addition, the serum zinc concentration in patients with primary SS was negatively correlated with both the ESSDAI score and subjective symptoms. Our observations demonstrated that serum zinc might influence the inflammatory status that was associated with the function of helper T cells or B cells in patients with primary SS. Future investigations into the effect of zinc in the pathogenesis of primary SS are warranted.

## Figures and Tables

**Table 1:** The clinical characteristics of the patients with pSS.

	Primary SS (*n* = 164)
Gender (women/men)	157/7
Age (years)	59.3 ± 14.9
Xerostomia	86.6% (142/164)
Xerophthalmia	76.4% (126/164)
Positive Saxon test	100.0% (1/1)
Positive Gum test	88.5% (123/139)
Positive Salivary gland scintigraphy	85.6% (119/139)
Positive Shirmer’s test	69.9% (102/146)
Positive Fluorescein staining	61.7% (82/133)
WBC (/µL)	4722.0 ± 1416.1
Neutrophil (/µL)	2723.6 ± 1223.5
Lymphocyte (/µL)	1461.4 ± 506.7
Hb (g/dL)	12.5 ± 1.3
Plt. (×10^3^/µL)	207.9 ± 6.3
Anti-Ro/SS-A antibody	85.4% (140/164)
Anti-La/SS-B antibody	42.1% (69/164)
Minor salivary gland biopsy (focus score ≥ 1)	82.3% (102/124)
Zinc concentration (µmol/L)	67.4 ± 10.2

SS: Sjögren’s syndrome; WBC: white blood cell count; Neutrophil: neutrophil count; Lymphocyte: lymphocyte count; Hb: hemoglobin; Plt: platelet count. The focus score was calculated as the focus number per 4 mm^2^.

**Table 2:** Study group characteristics.

	Primary SS (*n* = 164)	RA (*n* = 144)	
Sex (males/females)	7/157	25/119	*p* < 0.001
Age (years)	59.3 ± 14.9	64.9 ± 16.5	*p* = 0.05
Zinc concentration (µmol/L)	67.4 ± 10.2	72.4 ± 10.0	*p* = 0.007

SS: Sjögren’s syndrome. RA: rheumatoid arthritis. Ages and zinc. Concentrations are shown as the means ± standard deviation.

**Table 3:** Comparison of mean zinc concentration in the serum of patients with primary SS versus RA and health checkup recipients.

Zinc Concentration (µg/dL)	Primary SS (*n* = 157)	RA (*n* = 119)	Health Checkup Recipients (*n* = 883)
≥80	10.8% ab	22.7% a	61.0%
60 ≤ Zn < 80	63.1% ac	69.0% a	38.4%
<60	26.1% ad	7.6% a	0.6%

SS: Sjögren’s syndrome. RA: rheumatoid arthritis. Health checkup recipients’ [25]. *p*-values were obtained via Chi^2^ test. *p* < 0.05: significant. a: *p* < 0.001 compared with the health checkup recipient group. b: *p* = 0.012 compared with the RA group. c: *p* = 0.37 compared with the RA group. d: *p* < 0.001 compared with the RA group.

**Table 4:** Comparison of mean zinc concentrations of patients with primary SS with different disease activities.

	Zinc Concentration in Serum
ESSDAI < 5 (*n* = 117)	69.7 ± 102 (µg/dL)
ESSDAI ≥ 5 (*n* = 47)	60.6 ± 7.3 (µg/dL) *

SS: Sjögren’s syndrome. ESSDAI: European League Against Rheumatism Sjögren’s Syndrome Disease Activity Index. *p*-values were determined via Mann–Whitney *U*-test. * Significantly different at *p* < 0.001 vs. the ESSDAI < 5 group.

## Data Availability

The data presented in this study are available on request from the corresponding author.

## Supplementary Materials

The following supporting information can be downloaded at: https://www.mdpi.com/article/10.3390/jcm13164672/s1, Table S1: Original Questionnaire.
