# Supplementary material for: Subjective Symptoms and Disease Activity Related to Serum Zinc Concentration in Primary Sjögren’s Syndrome
Source: J Clin Med. 2024 Aug 9;13(16):4672. doi: 10.3390/jcm13164672 (PMC11355886; doi:10.3390/jcm13164672)
Supplement: Supplementary file 1 [file jcm-13-04672-s001.zip › jcm-3023784-supplementary.pdf]

Supplemental Table S1.Original questionnaire

If there are no symptoms below 0, if there are severe symptoms below 10, please put a check mark in the □.

mild symptoms ←degree of symptoms→ severe symptoms

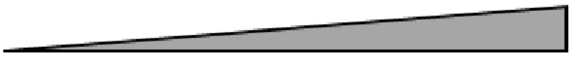

no symptoms most severe symptoms

	0	1	2	3	4	5	6	7	8	9	10
・dry mouth	□	□	□	□	□	□	□	□	□	□	□
	0	1	2	3	4	5	6	7	8	9	10
・prone to oral ulcers	□	□	□	□	□	□	□	□	□	□	□
	0	1	2	3	4	5	6	7	8	9	10
・pain in the tongue	□	□	□	□	□	□	□	□	□	□	□
	0	1	2	3	4	5	6	7	8	9	10
・difficulty in tasting	□	□	□	□	□	□	□	□	□	□	□
	0	1	2	3	4	5	6	7	8	9	10
・difficulty smelling	□	□	□	□	□	□	□	□	□	□	□
	0	1	2	3	4	5	6	7	8	9	10
・appetite loss	□	□	□	□	□	□	□	□	□	□	□
	0	1	2	3	4	5	6	7	8	9	10
・prone to infections	□	□	□	□	□	□	□	□	□	□	□
	0	1	2	3	4	5	6	7	8	9	10
・hair falls out	□	□	□	□	□	□	□	□	□	□	□
	0	1	2	3	4	5	6	7	8	9	10
・wound healing delay	□	□	□	□	□	□	□	□	□	□	□
	0	1	2	3	4	5	6	7	8	9	10
・prone to dermatitis	□	□	□	□	□	□	□	□	□	□	□
